# New Appliances and Things Medical

**Published:** 1900-01-27

**Authors:** 


### NEW APPLIANCES AND THINGS MEDICAL.

[We shall be glad to receive, at our Office, 28 & 29, Southampton Street, Strand, London, W.C., from the manufacturers, specimens of all new preparations and appliances which may be brought out from time to time.]

### CONSOMMÉ DE VOLAILLE AND CLEAR TURTLE SOUP.

(V. Benoist, 36, Piccadilly, W.)

With a view to their use as invalid specialities we have examined samples of the above prepared soups. The consommé is a finely-flavoured and beautifully cleared example of soup jelly. It is probably infinitely superior to anything of the kind that could be prepared at home, and should be highly acceptable as an addition to the ordinary invalid dietary. Variety is a matter of the greatest importance in the selection of invalid dainties, and for one change we highly recommend Benoist's Consommé de Volaille to the notice of those who have to cater for the capricious and difficult tastes of invalids and convalescents. The clear turtle is a fine specimen of this most nutritious soup. The sample which we have before us has been sent out in an earthenware pot with parchment cover. Although in itself a highly satisfactory method for so doing, being entirely free from contact with metal, the utmost care is necessary in observing definite rules for sterilising the contents, as well as the jar and its cover. In the specimen which we have examined there are several separate and distinct colonies of common mould, showing that sufficient care was not employed in putting up the soup in the preserving chamber.

### OROCO SOX.

(The Oroco Manufacturing Company, Wigan, Lancashire.)

As a substitute for the ordinary cork soles to be placed inside slipper or boot the Oroco Sox is a most ingenious and practical invention. The soles are cut out of Oroco paper, which is now the most popular form of corrugated packing paper; they are cut in several sizes, and can be readily pared down with scissors to the exact size. They are very cheap, and can frequently be secured at insignificant outlay. Being highly elastic and springy they are exceedingly comfortable, and should be a great comfort to those whose vocation in life necessitates long hours of standing. In the case of nurses, shop assistants, and servants they will prove a veritable boon.

